# Global process-based characterization factors of soil carbon depletion for life cycle impact assessment

**DOI:** 10.1038/s41597-021-01018-2

**Published:** 2021-09-09

**Authors:** Ricardo F. M. Teixeira, Tiago G. Morais, Tiago Domingos

**Affiliations:** MARETEC – Marine, Environment and Technology Centre, LARSyS, Instituto Superior Técnico, Universidade de Lisboa, 1049-001 Lisbon, Portugal

**Keywords:** Environmental sciences, Environmental impact

## Abstract

Regionalization of land use (LU) impact in life cycle assessment (LCA) has gained relevance in recent years. Most regionalized models are statistical, using highly aggregated spatial units and LU classes (e.g. one unique LU class for cropland). Process-based modelling is a powerful characterization tool but so far has never been applied globally for all LU classes. Here, we propose a new set of spatially detailed characterization factors (CFs) for soil organic carbon (SOC) depletion. We used SOC dynamic curves and attainable SOC stocks from a process-based model for more than 17,000 world regions and 81 LU classes. Those classes include 63 agricultural (depending on 4 types of management/production), and 16 forest sub-classes, and 1 grassland and 1 urban class. We matched the CFs to LU elementary flows used by LCA databases at country-level. Results show that CFs are highly dependent on the LU sub-class and management practices. For example, transformation into cropland in general leads to the highest SOC depletion but SOC gains are possible with specific crops.

## Abstract (summary)

**Table:** 

Measurement(s)	soil organic carbon depletion
Technology Type(s)	process-based modelling
Factor Type(s)	land use class • world region • soil carbon
Sample Characteristic - Environment	soil
Sample Characteristic - Location	global

Machine-accessible metadata file describing the reported data: 10.6084/m9.figshare.15163911

## Background & Summary

Land use (LU) and LU change are important drivers of change in the state of ecosystems globally^1^. Life cycle assessment (LCA) is increasingly used for estimating, comparing and highlight potential areas to reduce environmental impact of products and commodities throughout their supply chain^2–5^. Life cycle inventories (LCI) compile elementary flows, which are resources required in a unit process and emissions into the environment after production. Areas occupied and transformed, measured in m^2^.year and m^2^, respectively, are two of those LCI flows. In the last decade, different models were proposed to classify and characterize LCI flows into impacts, through life cycle impact assessment (LCIA). LCIA uses characterization factors (CFs) to determine the contribution of each inventory flow to each environmental indicator of interest. Soil organic carbon (SOC) depletion has been one of the most used indicators related to LU and LU change (among others, as biodiversity loss^6^) because it is a good proxy for LU damages to the biotic primary production potential of soils^7^ and other ecosystem services^8,9^. SOC depletion is included in environmental effects connected with the area of protection of “Natural Environmental”^1^. The Joint Research Centre of the European Commission recommends SOC depletion as the indicator for midpoint LU impacts^10^.

All published methods that used SOC depletion indicator are proxy-based and are based on a combination of statistical analysis and geographical information systems. They have varying levels of regionalization (i.e. spatial differentiation) and LU class differentiation. Among global methods, the first widely accepted method that proposed CFs was developed by Milà i Canals *et al*.^11^, a method without regionalization, i.e., for the same LU, a single CF is used globally. Other methods introduced regionalization at different levels. For example, Brandão and Milà i Canals^12^ developed CFs at the climate region scale and Teixeira *et al*.^13^ used a combination of climate region and soil type. Nevertheless, the number of regions and LU classes of these models is limited. For example, Teixeira *et al*.^13^ considered 96 regions and 4 LU classes. This is a consequence of requiring actual SOC measurements that need to be aggregated for statistical representativeness at wider geospatial scales and broader LU class.

Process-based modelling (PBM) is an approach based on formulating biogeochemical processes in to mathematical-ecological theory. These models consider site soil conditions, soil management practices and climatic data^14,15^. They consider temporal and spatial scales based on scenarios that characterize intra and inter-annual dynamics. They generally require more data than proxy-based models, but allow higher level of detail and have the possibly of reducing uncertainties because they are based on processes and not on statistics^16^. For example, the Rothamsted Carbon (RothC) Model^17^ is a well-accepted soil process model that simulates SOC turnover^18–21^. PBM have been used before to obtain CFs with higher level of regionalization and number of LU classes, but those were local/regional models only or involved only one type of LU systems (e.g. cropland)^22–25^.

Here, we propose a set of LU-LCIA CFs using SOC depletion as an indicator, using recently published data by Morais *et al*.^26^ involving global highly-regionalized and LU-specific results, from a global application of RothC. We considered 81 foreground LU classes (63 individual cropland classes, 16 forest classes and 1 grassland class, plus an urban LU class) and 17,203 regions. This is a new paradigm for how global CFs in LCIA can be calculated that combines PBM with LCA. Data resulting from this paper will enable LCA practitioners increased accuracy for their LCA studies in the “Natural Environmental” area of protection^1^, and will serve as demonstration that it is possible to use PBM globally and for all useful LU classes.

In this paper, we use specific terminology to separate two distinct methods for referring to the land transformation CFs calculated. We refer to “foreground” and “background” CFs for LU impacts. Background transformation CFs are equivalent to the “traditional” formulation used in the LCA community, i.e. CFs are defined with an unknown initial LU and a known final LU (e.g. “transformation to cropland”). The term “background” is due to the fact that these factors are mostly useful in combination with background LCI processes, as databases typically only include the final LU state and not the initial state prior to transformation. We define foreground CFs as those that have two known LU classes, i.e. when both initial and final LU classes are known (e.g. “transformation from irrigated tomato to irrigated cabbage”).

## Methods

### The RothC model

The Rothamsted Carbon Model (RothC) estimates carbon turnover in non-waterlogged soils^17^. It was developed for arable soils in the United Kingdom, but it has been expanded and successfully applied to model soil carbon dynamics also in grassland^18,27^ and forestry^28,29^ LUs in other regions of the World. It takes into account the effects of temperature, moisture content and soil type. SOC is divided in five compartments or pools, depending on decomposability: inert organic matter, easily decomposable plant material, resistant plant material, microbial biomass and humified organic matter. The inert organic matter pool is resistant to decomposition and does not receive C inputs^30^. Each compartment, except inert organic matter, decomposes according to a first-order decomposition process. The model uses a monthly step. Here, we used the RothC model to estimate the dynamics of SOC stock accumulation and loss after LU change.

### Land use characterization model

Here, we use the characterization model proposed by Milà i Canals *et al*.^31^ and updated by Koellner *et al*.^32^ with some modifications for transformation CFs. Land “occupation” and land “transformation” as basic types of land use elementary flows that affect ecosystem quality. Land occupation refers to the use of a given area for human purposes during a certain period, while land transformation refers to the conversion of a certain area to a new occupation.

The occupation CF, as defined by Koellner *et al*.^32^, is the difference between the attainable SOC (ASOC) for potential natural vegetation (PNV), designated as ASOC_PNV_, and the ASOC for LU2 (ASOC_LU2_). ASOC is the potential maximum SOC stored under a given LU given constant climate and soil conditions. PNV is the vegetation type that the LU system would revert to if human occupation ceased. The CF was calculated according to

1 $${{\rm{CF}}}_{{\rm{occup}}}\left[{\rm{t}}\;{\rm{C}}/{\rm{ha}}\right]={{\rm{ASOC}}}_{{\rm{PNV}}}-{{\rm{ASOC}}}_{{\rm{LU}}2}.$$

The model expressed by Eq. (1) assumes that the impact of occupying land are the foregone ecosystem services provided by SOC due to the fact that LU2 is delaying regeneration of land to PNV, measured as the difference in ASOC between LU2 and PNV.

**Fig. 1:**
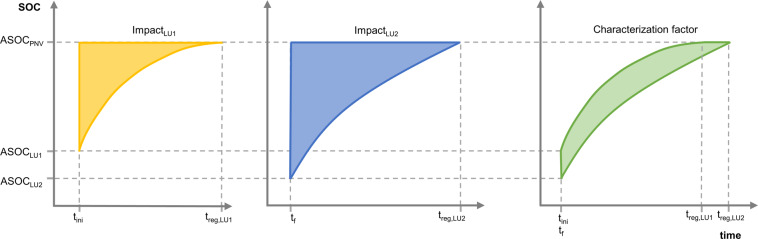
Graphical representations of the dynamic curves used in the calculation of land transformation characterization factors (where the factors are calculated using the areas shown in yellow, blue and green). ASOC_LU1_ – Attainable soil organic carbon content before transformation; ASOC_LU2_ - Attainable soil organic carbon content in the actual land use; ASOC_PNV_ - Attainable soil organic carbon content in natural vegetation; t_ini_ - the instant when the LU1 occupation ends; t_f_ - the instant when the LU2 occupation ends; t_reg,LU1_ - instant when SOC has reverted to the potential after LU1; t_reg,LU2_ - instant when SOC has reverted to potential after LU2; Impact_LU1-PNV_ – impact of transformation from LU1 to potential natural vegetation; Impact_LU2-PNV_ – impact of transformation from LU2 to potential natural vegetation.

For transformation, we considered an exponential transition (given by RothC) between the SOC of initial and final states while Koellner *et al*.^32^ considered a linear transition. The impact of transformation is the accumulated SOC deficit during revegetation with PNV between two cases – if regeneration started without transformation to LU2, and after occupation with LU_2_. The transformation CF is therefore the area comprised between the SOC curve during regeneration from LU_2_ and ASOC at PNV for the period between t_f_ and t_reg,LU2_ (Impact_LU2_ in Fig. 1), minus the area comprised between the SOC curve for regeneration from LU_1_ and the ASOC at PNV for the period between t_ini_ and t_reg,LU1_ (Impact_LU1_ in Fig. 1). ASOC is a characteristic of each LU type, and we assume that regeneration to PNV starts from LU systems in equilibrium (i.e. with SOC level at the start of the transition equal to ASOC). Occupation and transformation CFs express SOC depletion, which means that a positive CF implies higher SOC loss in the transition to LU_2_ (and vice-versa for a negative CF).

#### Foreground characterization factors

To calculate Impact_LU1_ and Impact_LU2_ (Fig. 1), we calculated the integral between ASOC at PNV and each SOC dynamic curve starting at the beginning of the transformation (t_ini_ and t_f_ for LU1 and LU2, respectively) and ending when ASOC is achieved (t_reg,LU1_ and t_reg,LU2_ for LU1 and LU2, respectively). In this approach both LUs are known, and therefore the CF is calculated according to

2 $$\begin{array}{l}{{\rm{CF}}}_{{\rm{transformation}}}[{\rm{t}}\;{\rm{C}}\cdot {\rm{year}}/{\rm{ha}}]={{\rm{Impact}}}_{{\rm{LU}}1}-{{\rm{Impact}}}_{{\rm{LU}}2}\\ =\,{{\rm{SOC}}}_{{\rm{PNV}}}-{\int }_{{{\rm{t}}}_{{\rm{ini}}}}^{+\infty }{{\rm{SOC}}}_{{\rm{LU}}1}({\rm{t}})\cdot {\rm{dt}}-{{\rm{SOC}}}_{{\rm{PNV}}}-{\int }_{{{\rm{t}}}_{{\rm{f}}}}^{+\infty }{{\rm{SOC}}}_{{\rm{LU}}2}({\rm{t}})\cdot {\rm{dt}}.\end{array}$$

#### Background characterization factors

For background transformation CFs LU1 is undetermined. Impact_LU1_ was calculated as the average of impacts of transformations from LU classes within each region according a LU map^33^. For example, if a certain region is divided in 50% cropland and 50% forest, Impact_LU1_ is the average impact of the individual crops feasible in that region multiplied by 50% plus the average impact of the individual forest types feasible in the region multiplied by 50%.

Here, we only calculated background transformation CFs at country-level because these CFs are meant to be used in background inventory flows that are at country-level (or even a higher level of aggregation).

### Data used

ASOC and SOC dynamic curves data was obtained from Morais *et al*.^26^ and is available in Zenodo^34^. Their work covers 80 LU classes, including 1 grassland class, 16 forest classes, and 63 agricultural classes. The 63 agricultural classes correspond to 28 individual crops the differ according to management practices. First, cropland SOC curves were determined for rainfed and irrigated production. Then, for cereal classes, they considered two management options for residue management: residues are left on the field and residues are removed from the field. All analyses were repeated for three organic fertilization scenarios.

We used the spatial aggregation proposed by Morais *et al*.^26^, where the world is divided in to 17,203 unique territorial units (UHTU). UHTUs are geographical regions where the local characteristics (i.e. soil type and texture, climate type and current LU) are uniform. Thus, UHTUs were obtained by overlaying thermal zones, land cover, soil type, soil texture and country. The UHTU map resolution is 0.083 decimal degrees (approximately 10 km × 10 km at equator) and is also available in Zenodo^34^.

LU maps used in the background transformation CFs were obtained from Erb *et al*.^35^ and can be downloaded from Erb *et al*.^36^. These maps consider four classes (cropland area, forestry area, grazing land and urban) for the all World. Resolution of all LU maps was also 0.083 decimal degrees. Each pixel has the fraction of each class of LU present (e.g., x% of cropland and y% of urban). The 63 agricultural LU classes from Morais *et al*.^26^ correspond to the cropland class, the 16 forest classes to the forestry, and the grassland class to the grazing land.

### Calculation procedure

First, we defined the PNV LU class (among forest and grassland classes) as a simplification that ASOC at PNV should be the maximum achievable ASOC in each UHTU, which was also the approach used by Teixeira *et al*.^13^. When the PNV was a forest and the initial SOC stock was significantly different from the initial SOC used by Morais *et al*.^26^, the fourth-degree polynomial obtained by Morais *et al*.^26^ led to implausible results. For example, using the parameters provided by Morais *et al*.^26^ for the forest growth period, if the initial SOC stock was significantly lower than the one used by Morais *et al*.^26^, the SOC stock after forest growth sometimes reached zero or even negative values. Thus, in order to correct for this issue, we ran the RothC model for the forest growth period in each UHTU for all possible transitions between forest and other LU classes. For the period between the end of the forest growth and SOC stabilization, we used the exponential fit from Morais *et al*.^26^. The initial SOC stock used for each LU class was the ASOC stock obtained by Morais *et al*.^26^. All the other input data required (soil, vegetation and climatic data) to use RothC was also the same used by Morais *et al*.^26^. Soils were characterized with the soil cover period, initial SOC stock and clay content. The soil cover period is a binary monthly variable, where 1 means that the soil was covered with vegetation during that month and 0 means that the soil was bare. The initial SOC stock was obtained from the European Soil Data Centre^37^. Clay content was obtained from the Harmonized World Soil Database^38^ (available from https://dare.iiasa.ac.at/44/). We used the IPCC methods^39–41^ and crop yields obtained from the Food and Agriculture Organization of the United Nations (FAO)^42^ to calculate C inputs from annual plant residues the residues. Precipitation was obtained from the database of the “Global Precipitation Climatology Project”^43^ and monthly average air temperature was obtained from MODIS^44^. Potential evapotranspiration was calculated using the Thornthwaite equation^45^, which uses monthly average air temperature, average day length, in hours, and number of days per month obtained from MODIS^44^.

We used a Monte Carlo method^46^ considering 100 unique set of SOC dynamic curves. For each SOC dynamic curve a different set of input parameters was used, i.e. for each LU class in a certain UHTU, RothC is run 100 times, and in each of the runs the climate data and soil inputs vary according to a normal distribution depicting intra-UHTU variability (see in detail in Morais *et al*.^47^). Thus, the final CFs, per LU and UHTU are equal to the average of the CFs obtained from the 100 runs. Sampling from a normal distribution ensured that the average results of all simulations were approximately equal to results obtained using the most representative data for each UHTU, while allowing for some outlier samples to be modelled, thus representing expected heterogeneity within each region.

Regarding transitions between the urban land use and PNV, we simulated this LU class in the RothC model by considering the soil covered all year and no carbon inputs in the soil, in 10 different UHTUs. After 100 years, there was almost no difference between the SOC stock and the inert organic matter pool (i.e. all other pools were close to zero) calculated by Morais *et al*.^26^ using the method by Weihermüller *et al*.^48^. Therefore, in each UHTU we set the initial SOC stock equal to the inert organic matter pool from Morais *et al*.^26^ and ran the RothC model to obtain the SOC dynamic curve between urban LU and the PNV. All the other input data required were also the same as used by Morais *et al*.^26^, using again a Monte Carlo approach^46^.

### Integration with LCI elementary flows

In most LCI databases, occupation and transformation flows are not at UHTU level or LU-specific. They are usually at country, continental or other representative scales and in aggregated LU classes. To ensure wide usability, we calculated occupation and transformation (for the background approach) CFs per country at aggregated LU classes for the elementary flows proposed by Koellner *et al*.^49^, which are used in the most common LCI databases (e.g. ecoinvent^50^ and GaBi^51^). CFs were aggregated at country level as the area-weighted average of all UHTUs in each country. LU aggregation was performed according the classification key shown in Table 1. Wetlands, bare areas and all water-related elementary flows do not have CFs for SOC depletion (as in other methods, e.g. Milà i Canals *et al*.^52^, which is the method used in the International Reference Life Cycle Data System - ILCD^10^), thus they were omitted from Table 1. We assumed that an “Unspecified” elementary flow has the highest CFs (i.e. CFs for urban LU class in this paper), except for “Unspecified, natural” where we considered the forest LU class with lowest ASOC (the highest CFs). All elementary flows related with human activities and unrelated with agriculture were assigned to the urban class in this paper. All grassland and pasture elementary flows were attributed to the grassland class in this paper. Agriculture-related elementary flows were mainly divided in crop type (annual crop/permanent crop) and different management practices (rainfed/irrigated).

**Table 1:** Classification key between elementary flows proposed by Koellner *et al*.^49^ and LU classes used in this paper.

ID	Elementary flows	LU class used in this paper
0	Unspecified	Urban
0.1	Unspecified, used	
0.2	Unspecified, natural	Forest class with lower attainable SOC stock
1	Forest	Average of all forest LU classes
1.1	Forest, natural	
1.1.1	Forest, primary	
1.1.2	Forest, secondary	
1.2	Forest, used	
1.2.1	Forest, extensive	
1.2.2	Forest, intensive	
3	Shrub land	Grassland
4	Grassland	
4.1	Grassland	
4.1.1	Grassland, natural	
4.1.2	Grassland, for livestock grazing	
4.2	Pasture/meadow	
4.2.1	Pasture/meadow, extensive	
4.2.2	Pasture/meadow, intensive	
5	Agriculture	Average of all croplands LU classes
5.1	Arable	Average of all annual crops LU classes
5.1.1	Arable, fallow	
5.1.2	Arable, non-irrigated	Average of all rainfed annual crops LU classes
5.1.2.1	Arable, non-irrigated, extensive	
5.1.2.2	Arable, non-irrigated, intensive	
5.1.3	Arable, irrigated	Average of all irrigated annual crops LU classes
5.1.3.1	Arable, irrigated, extensive	
5.1.3.2	Arable, irrigated, intensive	
5.1.4	Arable, flooded crops	Irrigated Rice
5.1.5	Arable, greenhouse	Average of all annual crops LU classes
5.1.6	Field margins/hedgerows	
5.2	Permanent crops	Average of all permanent crops LU classes
5.2.1	Permanent crops, non-irrigated	Average of all rainfed permanent crops LU classes
5.2.1.1	Permanent crops, non-irrigated, extensive	
5.2.1.2	Permanent crops, non-irrigated, intensive	
5.2.2	Permanent crops, irrigated	Average of all irrigated permanent crops LU classes
5.2.2.1	Permanent crops, irrigated, extensive	
5.2.2.2	Permanent crops, irrigated, intensive	
6	Agriculture, mosaic	Average of all croplands LU classes
7	Artificial areas	Urban
7.1	Urban	
7.1.1	Urban/industrial fallow	
7.1.2	Urban, continuously built	
7.1.3	Urban, discontinuously built	
7.1.4	Urban, green areas	
7.2	Industrial area	
7.3	Mineral extraction site	
7.4	Dump site	
7.5	Construction site	
7.6	Traffic area	
7.6.1	Traffic area, road network	
7.6.2	Traffic area, rail network	
7.6.3	Traffic area, rail/road embankment	

### Validation of characterization factors

We compared the results of this study with two proxy-based models that use SOC depletion or foregone carbon sequestration as the indicator for LU-LCIA and calculated CFs at global scale. The models are: Teixeira *et al*.^13^ (CFs can be downloaded from https://pubs.acs.org/doi/suppl/10.1021/acs.est.8b00721/suppl_file/es8b00721_si_002.xlsx) and Brandão and Milà i Canals^12^ (CFs downloaded from https://static-content.springer.com/esm/art%3A10.1007%2Fs11367-012-0381-3/MediaObjects/11367_2012_381_MOESM1_ESM.xlsx). In both models, croplands and forest LU classes are depicted as a single class. We compared the different methods in terms of regionalization (i.e. number of UHTUs), LU desegregation (i.e. number of LU classes) and the absolute value of CFs.

## Data Records

We calculated 370,760 foreground occupation CFs and 5,198,763 foreground transformation CFs. Online-only Table 1 presents the number of occupation and transformation foreground CFs per LU class. For transformation we only calculated one CF for each pair of LU classes, e.g. we only considered “transformation from X to Y” and not “transformation from Y to X”, because the latter CF can be obtained only multiplying the former CF by −1. The full list of occupation and transformation CFs is shown at Zenodo repository^53^.

Unlike most studies calculating CFs, we also quantified uncertainty for all CFs. This is the first paper that considers SOC depletion at global level that provides CFs uncertainty (mean and standard deviation).

In order to facilitate application by LCA practitioners, we additionally calculated occupation and transformation CFs using the background approach. In this approach the foreground CFs were aggregated per country and to the LU classes proposed by Koellner *et al*.^49^, which are used in the most common LCI databases (e.g. ecoinvent^50^ and GaBi^51^ databases). The key between LU classes used in this paper and elementary flows proposed by Koellner *et al*.^49^ is in Table 1 (in the Methods section). We calculated CFs for the 74 elementary flows proposed by Koellner *et al*.^49^. In total we calculated 9,464 background occupation and transformation CFs. Background CFs also have an associated mean and standard deviation.

### Comparison between land uses

**Fig. 2:**
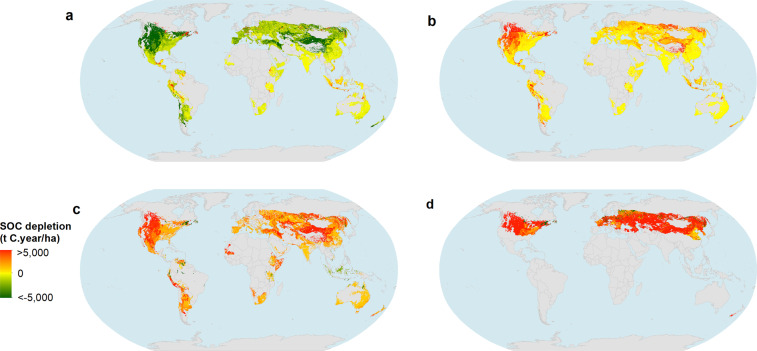
Graphical representation of characterization factors for transformation (**a**) from rainfed maize removing residues from the field to irrigated maize maintaining residues on the field, (**b**) from rainfed maize (maintaining residues on the field) to urban, (**c**) from grassland to rainfed maize (maintaining residues on the field), and (**d**) from needleleaf evergreen forest (in warm temperate and dry region) to rainfed maize (maintaining residues on the field). A positive value means a SOC depletion/loss (and conversely for SOC gain). SOC – Soil organic carbon.

Figure 2 shows the foreground transformation CF for four different transitions. The highest SOC depletions are found in the urban LU class (Fig. 2b). This occurs due to the fact that urban systems have no C inputs into the soil, and thus in the long term the ASOC stock is equal to the inert organic matter (in all other LU classes there are active organic matter pools). Among croplands, leaving crop residues on the field leads, in general, to an increase on SOC stock, and thus a negative CF when the initial LU is the same crop but without residues left on the field (Fig. 2a). Transitions to croplands from forest results in a SOC depletion/loss globally with rare exceptions (Fig. 2d). Transitions from grassland (Fig. 2c) lead, in general, to SOC depletion/loss, however for some crops under specific management practices (leaving residues on the field and irrigation) can result in SOC gains (negative SOC depletion) due to higher C input into the soil. An example of this is North American irrigated wheat maintaining residues on the field.

Among croplands, SOC depletion/gain is highly dependent on the specific crop type. For example, for irrigated maize, the effect of maintaining residues on the field results in a negative CF for about 87% of the UHTUs - Fig. 2a. SOC stock increases when cropland is converted to grassland in most of the UHTUs and initial LU classes. The exceptions are crop classes that have higher C inputs that grasslands, as is the case of cereals with high production of sub-products/residues that are incorporated into the soil. Results also illustrate that SOC dynamics are mostly influenced by crop residues and not precipitation and temperature, e.g. most of the UHTUs when transformed from rainfed maize removing residues from the field to irrigated maize maintaining residues on the field gain SOC, regardless of the region (Fig. 2a). This is even more evident when the final LU is urban (Fig. 2b), where in all UHTU the transformation leads to SOC losses. This means that the CFs are more affected by C inputs into the soil than mineralization rates (which depend on the climatic conditions). For example, less than 10% of the UHTs have positive CF for transformation to grassland from irrigated maize without residues left on the field (Fig. 2c). However, the percentage increases to 30% when the initial LU is irrigated maize with residues left on the field due the increase of C inputs in the soil.

On average, occupation CFs when crop residues are left on the field are 60% lower (less SOC depletion) than the CFs for the same crop when residues are removed. The difference is largest for wheat (80% less SOC depletion) and the smallest difference is for barley (48% less SOC depletion). The average effect of irrigation on all agricultural classes is a decrease of 30% in SOC depletion. The difference is minimum for sweet potato (less than 5% reduction) and maximum for maize (about 70% reduction). For both management practices, SOC depletion is affected the most in temperate regions, which is where crops have the highest potential yields and the highest need for irrigation. This result is a consequence of the ASOC stocks in Morais *et al*.^26^. For transformation CFs, the differences already found for occupation CFs are amplified due to the non-linearity of SOC regeneration, i.e. the difference between SOC curves between LU1 and LU2 are considered until the infinity (see in detail in the Methods section) while other models consider linear regeneration in finite time.

Most of the transformations from forest classes result in a SOC stock loss (positive transformation CF). Similar to the case of transformations from grassland, only few cases result in a negative transformation CF. For example, only 5% of the UHTUs have negative CFs for the transformation from broadleaf deciduous forest in the climate zone “warm temperate, dry” to irrigated maize without residues left on the field.

In general, forest LUs have higher uncertainty than agricultural LU classes, ±95 t c/ha and ±50 t C/ha, respectively (average SOC stock: 163 t C/ha and 43, respectively). “Needleleaf Evergreen - Cold temperate, dry” is the class with highest uncertainty for occupation CFs, i.e. average confidence interval ±200 t C/ha. “Needleleaf Evergreen - Tropical” is the LU class with the lowest interval of confidence (i.e. ±27 t C/ha). Among croplands, interval of confidence range between about 30 t C/ha (“Rainfed Olives”) and 120 t C/ha (“Irrigated Sugarcane”).

## Technical Validation

**Fig. 3:**
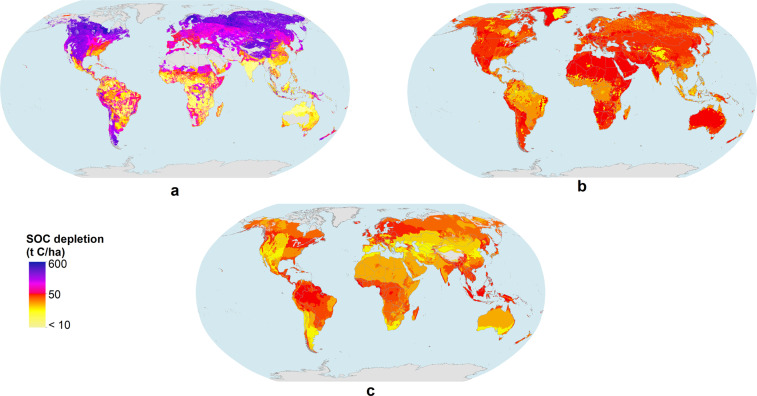
Occupation characterization factors (CFs) for the land class “agriculture/arable” elementary flow according to (**a**) CFs from this paper, (**b**) Teixeira *et al*.^13^ (consensus calculated by simple average), and (**c**) Brandão and Milà i Canals^12^. Maps depict the maximum resolution available in each study. SOC – Soil organic carbon.

Figure 3 presents occupation CFs for the “agriculture/arable” LU class for the background CFs obtained in this paper, and the comparable CFs from Teixeira *et al*.^13^ and Brandão and Milà i Canals^12^. Summary statistics about the aggregation of those factors at country scale are shown in Table 2. The geographic applicability of CFs is dependent on the number of UHTUs. CFs proposed in this paper used about 17,000 UHTU and therefore the variation range is the largest (between 30 and 600 t C/ha - Fig. 3a). Teixeira *et al*.^13^ used only 96 UHTUs (less than 0.5% of the total number of UHTUs used in this paper), and CFs range between 10 and 40 t C/ha (Fig. 3b depicts consensus CFs using simple average approach). Brandão and Milà i Canals^12^ used the lowest number of UHTUs, only 10 UHTUs, and CFs range between 7 and 30 t C/ha (Fig. 3c).

**Table 2:** Comparative statistics of country-level CFs for the main land use classes between the present study, Teixeira *et al*.^13^ (consensus calculated by simple average) and Brandão and Milà i Canals^12^.

LU class	This study		Teixeira *et al*.^13^		Brandão and Milá i Canals^12^	
	Mean	Stdev	Mean	Stdev	Mean	Stdev
Arable	154.57	95.51	23.13	2.78	23.47	6.35
Permanent crops	136.79	64.56				
Grassland	153.92	120.27	20.50	3.01	10.18	3.27
Urban	169.82	117.88	25.56	2.90	59.53	17.18
Forest	93.37	87.64	18.34	3.65	NaN	NaN

Despite these differences, in general the hotspots of SOC depletion are similar for all models. Highest SOC depletions are found at higher latitudes (i.e. North America and Northern Europe). There is a mismatch for specific countries such as India and Australia, which are hotspots of SOC loss in the work of Teixeira *et al*.^13^ and, to a less degree, Brandão and Milà i Canals^12^, but not in the CFs obtained in this paper. The main factors that explain differences are the variability in the data sources particularly for characterizing ASOC at PNV. Here, ASOC before and after regeneration are both calculated using the same model and therefore are quantitatively consistent. Other characterization models in the literature use different sources for quantifying SOC at PNV. ASOC at PNV is highly variable between sources. For example, the IPCC^39^ indicates that the maximum ASOC at PNV is 146 t C/ha (for volcanic soils and boreal climate region), while according to the Global Soil Organic Carbon Map^54^ from the FAO the maximum SOC stock is about 750 t C/ha in the boreal climate region. Another example of this is Brazil, one of countries with the highest occupation CF for Brandão and Milà i Canals^12^ but not in CFs proposed in this paper and in CFs proposed by Teixeira *et al*.^13^. Again, this is ultimately due to differences in the quantification of ASOC at PNV. The PNV LU class in Brazilian UHTUs is “Tropical forest”, which according to the IPCC^39^ has lower C inputs than other forest LU classes, which combined with high mineralization produces fast organic matter turnover and therefore lower ASOC stock.

## Usage Notes

One commonly referred problem regarding the use of advanced CFs from modelling is the fact there is insufficient resolution in inventories to use them^55^. This problem can be easily overcome for the CFs presented in this paper due to the distinction we introduced between background and foreground at LCIA level. At LCI level, it is already common to think in terms of background/foreground, but for LCIA the same distinction is useful. Put plainly, the idea is to apply aggregated/simplified CFs when there is no field-level data, and more specific CFs when there can be sufficient information. To use these CFs in an LCA study, we propose joint use of foreground and background CFs as they are compatible and were obtained consistently through the application of the same model and data sources. During data collection for the life cycle inventory (LCI) stage, data regarding LU occupation and transformation should be compiled (including information for the initial and final LU classes). In the LCIA stage, foreground CFs should be applied for foreground LU inventory flows. Background CFs should be applied for background LU elementary flows obtained from LCA databases (or other sources), which just describe LU occupation and transformation for aggregated LU classes (as Koellner *et al*.^49^). Following this procedure, practitioners will obtain more accurate impact assessment for the foreground processes/elementary flows (which usually represent most of the impact), instead of using the same highly generic CFs for both background and foreground elementary flows as is current practice in LCIA.

To facilitate the work of LCA practitioners, we provide all the CFs produced in this paper in a Zenodo repository^53^. This repository includes all the foreground and background CFs, in multiple data formats. All the original SOC dynamic curves used for calculating the CFs are also included in this Zenodo repository^53^ (including transitions for crop, grassland, forest and urban LU classes) in the file “SOC_dynamics.zip”.

The foreground occupation and transformation CFs (at UHTU level and at country level) are available in raster format (tiff file) and table format (excel file). The foreground occupation CFs in raster format are zipped in the file “Raster_Foreground_Occupation.zip” (one file per LU class) and foreground transformation CFs in raster format are zipped in the file “Raster_Foreground_Transformation.zip” (one file per LU class transition). The CFs available in raster format (tiff files) can be opened using geographic information systems (GIS) tools. Both foreground occupation and transformation CFs in table format are in the file “Table_CFs_foreground.xlsx” (all LU classes and transition in the same file).

The background occupation and transformation CFs (at country level) can also be downloaded in raster format (tiff file) and table format (excel file). The foreground occupation CFs in raster format are zipped in the file “Raster_Background_Occupation.zip” (one file per LU class) and transformation foreground CFs in raster format are zipped in the file “Raster_Background_Transformation.zip” (one file per LU transition). Both background occupation and transformation CFs in table format are in the file “Table_CFs_background.xlsx” (all LU classes and transition in the same file).

Finally, we also provide the background CFs produced in this paper in an OpenLCA^56^ impact assessment method file “LCIA_OpenLCA_file.zip”, available in the same Zenodo repository^53^. This file can be used without adaptation for the elementary flows in the PEF database (zip file compatible with JSON-LD).

## Data Availability

All code necessary to calculate the CFs is freely available from a Zenodo repository^57^. The ASOC and SOC data used to calculate CFs for crop LU classes were obtained from Morais *et al*.^34^ (Zenodo repository^34^). We used MATLAB release R2018a to calculate the CFs, including the new RothC runs for transitions to forests, which are available in Morais *et al*.^57^ (Zenodo repository). The script to run the MATLAB version of the RothC model is the script “RothC_TMorais.m”, and the script to calculate foreground and background CFs is “CFs_calculation.m”. The code is not commented, but detailed instructions for how to use the MATLAB scripts is in the Zenodo repository can be provided by the authors after e-mail contact. The Zenodo repository also indicates the list of data needed to run the model (references for collecting the data can be found throughout the paper). Morais *et al*.^53^ (Zenodo repository) includes all the SOC dynamics and obtained CFs.

## Online-only Table

**Online-only Table 1:** Summary of land use (LU) classes used in this paper and respective number of occupation and transformation characterization factors (CFs).

LU class used in this paper	Number of occupation CFs	Number of transformation foreground CFs
Irrigated Bananas	1,753	54,752
Rainfed Bananas	1,767	53,576
Irrigated Barley, residues left on the field	4,784	169,005
Rainfed Barley, residues left on the field	4,783	164,194
Irrigated Barley, residues removed from the field	4,713	157,738
Rainfed Barley, residues removed from the field	4,702	152,338
Irrigated Cabbages	6,924	188,426
Irrigated Carrots	6,589	176,808
Irrigated Oranges	6,556	162,372
Rainfed Oranges	6,596	156,178
Irrigated Coconuts	915	19,817
Rainfed Coconuts	916	18,919
Irrigated Coffee	497	11,042
Rainfed Coffee	526	10,996
Irrigated cotton	2,941	84,648
Rainfed cotton	2,942	81,620
Irrigated Groundnuts	1,492	43,516
Rainfed Groundnuts	1,492	42,024
Irrigated Maize, residues left on the field	5,333	141,139
Rainfed Maize, residues left on the field	5,333	135,806
Irrigated Maize, residues removed from the field	5,217	128,451
Rainfed Maize, residues removed from the field	5,200	122,815
Irrigated Oil palm	73	1,197
Rainfed Oil palm	73	1,124
Irrigated Onions	2,827	64,054
Irrigated Potatoes	11,039	215,262
Rainfed Potatoes	11,038	204,251
Irrigated Rapeseed, residues left on the field	4,091	89,783
Rainfed Rapeseed, residues left on the field	4,090	85,665
Irrigated Rapeseed, residues removed from the field	4,091	81,611
Rainfed Rapeseed, residues removed from the field	4,089	77,495
Irrigated Rice	5,078	92,565
Rainfed Rice	5,078	87,487
Irrigated Sorghum, residues left on the field	5,900	101,426
Rainfed Sorghum, residues left on the field	5,907	95,584
Irrigated Sorghum, residues removed from the field	5,367	84,038
Rainfed Sorghum, residues removed from the field	5,446	79,106
Irrigated Soybeans	5,551	93,852
Rainfed Soybeans	5,551	88,297
Irrigated Sugar beet	3,066	49,334
Rainfed Sugar beet	3,066	46,272
Irrigated Sugarcane	4,008	49,229
Rainfed Sugarcane	4,022	48,808
Irrigated Sunflower	4,757	72,568
Rainfed Sunflower	4,717	67,356
Irrigated Sweet potatoes	7,817	88,884
Rainfed Sweet potatoes	7,814	81,060
Irrigated Tobacco	9,900	103,519
Rainfed Tobacco	9,904	93,507
Irrigated Tomatoes	11,980	102,953
Rainfed Tomatoes	11,977	90,985
Irrigated Wheat, residues left on the field	7,912	74,389
Rainfed Wheat, residues left on the field	7,912	66,487
Irrigated Wheat, residues removed from the field	7,821	58,056
Rainfed Wheat, residues removed from the field	7,761	49,829
Irrigated Cocoa	268	1,357
Rainfed Cocoa	279	1,154
Irrigated Grapes	4,821	29,034
Rainfed Grapes	4,830	24,249
Irrigated Olives	231	1,639
Rainfed Olives	235	1,458
Irrigated Apples	4,361	19,153
Rainfed Apples	4,384	14,856
Broadleaf Deciduous - Boreal, dry	1,193	3,749
Needleleaf Evergreen - Boreal, dry	1,191	2,647
Broadleaf Deciduous - Boreal, moist	1,176	1,152
Needleleaf Evergreen - Boreal, moist	1,147	775
Broadleaf Deciduous - Cold temperate, dry	4,828	24,019
Needleleaf Evergreen - Cold temperate, dry	4,861	18,402
Broadleaf Deciduous - Cold temperate, moist	4,945	16,994
Needleleaf Evergreen - Cold temperate, moist	4,919	4,701
Broadleaf Deciduous - Warm temperate, dry	4,811	9,456
Needleleaf Evergreen - Warm temperate, dry	4,878	6,357
Broadleaf Deciduous - Warm temperate, moist	3,106	10,546
Needleleaf Evergreen - Warm temperate, moist	3,106	0
Broadleaf Deciduous – Subtropical	3,106	7,442
Needleleaf Evergreen – Subtropical	3,106	4,338
Broadleaf Deciduous – Tropical	7,888	9,282
Needleleaf Evergreen – Tropical	7,888	919
Grassland	5,755	5,668
Urban	1,753	17,203

The column “Number of occupation CFs” includes all the regions where each LU is possible. The “Number of transformation foreground CFs” includes CFs “transformation from LU_1_ to LU_2_” and “transformation from LU_2_ to LU_1_”.

## References

[1] Vidal Legaz B (2017). Soil quality, properties, and functions in life cycle assessment: an evaluation of models. J. Clean. Prod..

[2] Souza DM, Teixeira RFM, Ostermann OP (2015). Assessing biodiversity loss due to land use with Life Cycle Assessment: are we there yet?. Glob. Chang. Biol..

[3] Poore J, Nemecek T (2018). Reducing food’s environmental impacts through producers and consumers. Science.

[4] Hellweg S, Milà i Canals L (2014). Emerging approaches, challenges and opportunities in life cycle assessment. Science.

[5] Morais TG, Teixeira RF, Domingos T (2017). A step toward regionalized scale-consistent agricultural life cycle assessment inventories. Integr. Environ. Assess. Manag..

[6] Chaudhary A, Brooks TM (2018). Land Use Intensity-Specific Global Characterization Factors to Assess Product Biodiversity Footprints. Environ. Sci. Technol..

[7] Bot, A. & Benites, J. *The importance of soil organic matter: key to drought resistant soil and sustained food and production*. (2005).

[8] Garrigues E, Corson MS, Angers DA, van der Werf HMG, Walter C (2012). Soil quality in Life Cycle Assessment: Towards development of an indicator. Ecol. Indic..

[9] Millenium Ecosystem Assessment. *Ecosystems and Human Well-Being: Synthesis*. *Ecosystems* (2005).

[10] European Commission - Joint Research Centre & Institute for Environment and Sustainability. *International Reference Life Cycle Data System (ILCD) Handbook - Recommendations for Life Cycle Impact Assessment in the European Context*. *First edition November 2011* (Publications Office of the European Union, 2011).

[11] Milà i Canals, L. Contributions to LCA methodology for agricultural systems. Site-dependency and soil degradation impact assessment. Dissertation. (Universitat Autònoma de Barcelona, 2003).

[12] Brandão M, Milà i Canals L (2013). Global characterisation factors to assess land use impacts on biotic production. Int. J. Life Cycle Assess..

[13] Teixeira RFM, Morais TG, Domingos T (2018). Consolidating Regionalized Global Characterization Factors for Soil Organic Carbon Depletion Due to Land Occupation and Transformation. Environ. Sci. Technol..

[14] Luo Y, Keenan TF, Smith M (2015). Predictability of the terrestrial carbon cycle. Glob. Chang. Biol..

[15] Cuddington K (2013). Process-based models are required to manage ecological systems in a changing world. Ecosphere.

[16] Othoniel B, Rugani B, Heijungs R, Benetto E, Withagen C (2016). Assessment of Life Cycle Impacts on Ecosystem Services: Promise, Problems, and Prospects. Environ. Sci. Technol..

[17] Coleman K (1997). Simulating trends in soil organic carbon in long-term experiments using RothC-26.3. Geoderma.

[18] Morais TG, Teixeira RFM, Rodrigues NR, Domingos T (2018). Characterizing livestock production in Portuguese sown rainfed grasslands: Applying the inverse approach to a process-based model. Sustainability.

[19] Smith J (2007). Projected changes in the organic carbon stocks of cropland mineral soils of European Russia and the Ukraine, 1990–2070. Glob. Chang. Biol..

[20] Cerri CEP (2007). Predicted soil organic carbon stocks and changes in the Brazilian Amazon between 2000 and 2030. Agric. Ecosyst. Environ..

[21] Gottschalk P (2012). How will organic carbon stocks in mineral soils evolve under future climate? Global projections using RothC for a range of climate change scenarios. Biogeosciences.

[22] Morais TG, Domingos T, Teixeira RFM (2016). A spatially explicit life cycle assessment midpoint indicator for soil quality in the European Union using soil organic carbon. Int. J. Life Cycle Assess..

[23] Boone L (2018). Accounting for the impact of agricultural land use practices on soil organic carbon stock and yield under the area of protection natural resources - Illustrated for Flanders. J. Clean. Prod..

[24] Morais TG (2018). A proposal for using process-based soil models for land use Life cycle impact assessment: Application to Alentejo, Portugal. J. Clean. Prod..

[25] Sevenster M, Luo Z, Eady S, Grant T (2019). Including long-term soil organic carbon changes in life cycle assessment of agricultural products. Int. J. Life Cycle Assess..

[26] Morais, T. G., Teixeira, R. F. M. & Domingos, T. Some croplands can potentially accumulate more soil carbon than forests and grasslands: Implications of detailed global modelling. *PLoS One* (2019). 10.1371/journal.pone.0222604 PMC6752864 31536571

[27] Liu DL, Chan KY, Conyers MK, Li G, Poile GJ (2011). Simulation of soil organic carbon dynamics under different pasture managements using the RothC carbon model. Geoderma.

[28] Rumpel C, Balesdent J, Grootes P, Weber E, Kögel-Knabner I (2003). Quantification of lignite- and vegetation-derived soil carbon using 14C activity measurements in a forested chronosequence. Geoderma.

[29] Hashimoto S, Wattenbach M, Smith P (2011). Litter carbon inputs to the mineral soil of Japanese Brown forest soils: Comparing estimates from the RothC model with estimates from MODIS. J. For. Res..

[30] Kutsch, W. L., Bahn, M. & Heinemeyer, A. *Soil Carbon Dynamics: An Integrated Methodology*. (Cambridge University Press, 2009).

[31] Milà i Canals L (2007). Key Elements in a Framework for Land Use Impact Assessment Within LCA. Int. J. Life Cycle Assess..

[32] Koellner T (2013). UNEP-SETAC guideline on global land use impact assessment on biodiversity and ecosystem services in LCA. Int. J. Life Cycle Assess..

[33] NASA LP DAAC. Land Cover Type Yearly L3 Global 0.05Deg CMG (MCD12C1). *NASA EOSDIS Land Processes DAAC, USGS Earth Resources Observation and Science (EROS) Center* https://lpdaac.usgs.gov/dataset_discovery/modis/modis_products_table/mcd12c1 (2017).

[34] Morais TG, Teixeira RFM, Domingos T (2019). Zenodo.

[35] Erb K-HK (2007). A comprehensive global 5 min resolution land-use data set for the year 2000 consistent with national census data. J. Land Use Sci..

[36] Erb, K.-H. K. *et al*. Data Download: A comprehensive global 5 min resolution land-use data set for the year 2000 consistent with national census data. https://boku.ac.at/fileadmin/data/H03000/H73000/H73700/Data_Download/Data/Land_Use_Download_as_package.zip (2020).

[37] ESDAC. Global Soil Organic Carbon Estimates. http://esdac.jrc.ec.europa.eu/content/global-soil-organic-carbon-estimates (2012).

[38] FAO, IIASA, ISRIC, ISSCAS & JRC. *Harmonized World Soil Database (version 1.2)*. (2012).

[39] IPCC. *2006 IPCC Guidelines for National Greenhouse Gas Inventories. Institute for Global Environmental Strategies (IGES) for the Intergovernmental Panel on Climate Change*. (2006).

[40] IPCC. *Revised 1996 IPCC Guidelines for National Greenhouse Gas Inventories. Volume 2 - Workbook*. (1997).

[41] IPCC. *Good Practice Guidance for Land Use, Land-Use Change and Forestry. Institute for Global Environmental Strategies (IGES) for the Intergovernmental Panel on Climate Change*. (2003).

[42] FAO. *Food and Agriculture Organization of the United Nations - Statistics Division*. http://faostat.fao.org/ (2018).

[43] NASA. *Global Precipitation Analysis*. https://gpm.nasa.gov/resources/documents/precipitation-processing-system-pps-transition-ftp-ftps-gpm-research-production (2021).

[44] DAAC, L. MODIS/Terra Land Surface Temperature and Emissivity Monthly L3 Global 0.05Deg CMG. https://lpdaac.usgs.gov/dataset_discovery/modis/modis_products_table/mod11c3 (2016).

[45] Thornthwaite CW (1948). An Approach toward a Rational Classification of Climate. Geogr. Rev..

[46] Metropolis N, Ulam S (1949). The Monte Carlo method. J. Am. Stat. Assoc..

[47] Morais TG, Teixeira RFM, Domingos T (2019). Detailed global modelling of soil organic carbon in cropland, grassland and forest soils. PLoS One.

[48] Weihermüller L, Graf A, Herbst M, Vereecken H (2013). Simple pedotransfer functions to initialize reactive carbon pools of the RothC model. Eur. J. Soil Sci..

[49] Koellner T (2013). Principles for life cycle inventories of land use on a global scale. Int. J. Life Cycle Assess..

[50] Wernet G (2016). The ecoinvent database version 3 (part I): overview and methodology. Int. J. Life Cycle Assess..

[51] Thinkstep. GaBi Database website. http://www.gabi-software.com/databases/gabi-databases/ (2018).

[52] Milà i Canals, L., Muñoz, I., McLaren, S. & Brandão, M. *LCA Methodology and Modelling Considerations for Vegetable Production and Consumption*. http://www.ces-surrey.org.uk/ (2007).

[53] Morais TG, Teixeira RFM, Domingos T (2020). Zenodo.

[54] FAO and ITPS. *Global Soil Organic Carbon Map (GSOCmap) Version 1.5*. 10.4060/ca7597en (FAO, 2020).

[55] Teixeira RFM, Morais TG, Domingos T (2018). A practical comparison of regionalized land use and biodiversity life cycle impact assessment models using livestock production as a case study. Sustain..

[56] GreenDelta. openLCA v1.10.3. *GreenDelta Berlin* (2020).

[57] Morais TG, Teixeira RFM, Domingos T (2020). Zenodo.

